# Supplementary material for: Malignant Hyperthermia: An Anesthesiology Simulation Case for Early Anesthesia Providers
Source: MedEdPORTAL. 2017 Mar 7;13:10550. doi: 10.15766/mep_2374-8265.10550 (PMC6342051; doi:10.15766/mep_2374-8265.10550)
Supplement: Supplementary file 1 — A. Simulation Case.docx B. Critical Actions.docx C. Debriefing Materials.docx D. Pre Post Test.docx E. Simulation Course Evaluation.docx [file mep-13-10550-s001.zip › _Educational Summary Report_10550.pdf]

MedEdPORTAL®
The Journal of Teaching and Learning Resources

Crossref

Original Publication

OPEN ACCESS

Malignant Hyperthermia: An Anesthesiology Simulation Case for Early Anesthesia Providers

Johnny Quick, MD, Rachana Murthy, Nitin Goyal, MD*, Steven Margolis, MD, Gregory Pond, MD, Kimberly Jenkins, MD
*Corresponding author: nitin.goyal@utoledo.edu

Citation: Quick J, Murthy R, Goyal N, Margolis S, Pond G, Jenkins K. Malignant hyperthermia: an anesthesiology simulation case for early anesthesia providers. *MedEdPORTAL*. 2017;13:10550. https://doi.org/10.15766/mep_2374-8265.10550

Copyright: © 2017 Quick et al. This is an open-access article distributed under the terms of the Creative Commons Attribution license.

Appendices

A. Simulation Case.docx
B. Critical Actions.docx
C. Debriefing Materials.docx
D. Pre Post Test.docx
E. Simulation Course Evaluation.docx

All appendices are peer reviewed as integral parts of the Original Publication.

Abstract

Introduction: The patient is a 40-kg, 12-year-old Caucasian male with history of asthma who is undergoing an elective inguinal hernia repair. There is no family history of anesthesia-related complications. The surgery proceeds under general anesthesia with an IV induction with propofol, fentanyl, and succinylcholine; intubation under direct laryngoscopy; and maintenance with isoflurane. During the surgery, he develops malignant hyperthermia (MH). **Methods:** Learners are to identify the signs of MH, including tachycardia, hypercapnia, muscle rigidity, and renal failure, and provide the appropriate treatment, resuscitation, and follow-up care. Anesthesiology faculty in the room assist and offer guided instruction to aid the learners in achieving these goals. **Results:** The simulation was completed by 24 medical students with 2 weeks of anesthesia training and daily lectures on various anesthesia topics. Verbal feedback from the learners was positive, and many appreciated the preparation in how to prioritize the management of such a rare but life-threatening anesthesia emergency. Based on reviewers' recommendations, a learner evaluation of the session and pre- and posttest exams have been developed but have not yet been used with learners. **Discussion:** The simulation not only was received well by the students but was also crucial to understanding the benefits of simulation training in the field of anesthesiology, especially when rare diseases are difficult to encounter in real life. Future simulations will incorporate other rare but important disease processes in the simulation training environment to allow anesthesia providers to learn in a safe setting without detriment to any patient.

Keywords

Simulation, Anesthesiology, Malignant Hyperthermia

Educational Objectives

By the end of the module, the learner will be able to:

1. Identify and treat malignant hyperthermia (MH) in the intraoperative setting in an organized team.
2. Discuss differential diagnosis of MH.
3. Identify and understand the chronological order of developing MH, including end-tidal carbon dioxide, heart rate, temperature, and muscle rigidity.
4. Identify the change in blood tests, including arterial blood gas, electrolyte panel, and renal function.
5. Apply the algorithm for treatment of MH, including dantrolene administration, with an emphasis on teamwork.
6. Recognize the patient's response to appropriate (and inappropriate) management interventions.
7. Develop individual and team skills in management of MH.
8. Develop team-oriented communication.
9. Develop a method to allocate tasks efficiently within a group.

Introduction

Any patient may potentially develop malignant hyperthermia (MH) intraoperatively. We aimed this simulation curriculum to educate early anesthesia providers in the accurate identification of and appropriate treatment regimen for this disease process. The target audience includes medical students, Postgraduate Year 1 residents, Clinical Anesthesia 1 residents, and student nurse anesthetists. The simulation can be adapted for senior residents, faculty, and mid-level anesthesia providers, but such modifications are beyond the scope of this publication. The prerequisite knowledge and skills needed are a fundamental understanding of MH as a disease process and of the signs and symptoms of the disease intraoperatively, as well as the ability to reconstitute and draw a powdered form of medication for administration.

We chose a simulation setting in an intraoperative environment with an anesthetized patient to give learners the most realistic presentation of MH. The learners can identify specific signs and symptoms that are clear indicators the patient has developed MH. Then, they can immediately start the required treatment and resuscitation. At our institution, learners were asked to familiarize themselves with the Malignant Hyperthemia Association of the United States website and its various tools.

Methods

The case is fully presented for facilitators in the simulation case file (Appendix A). A separate critical actions checklist (Appendix B) is also included for learners to reference while running the simulation. A debriefing handout (Appendix C) is included to facilitate the postassessment session. Prior to the simulation, learners are asked to read an article by Jurkat-Rott, McCarthy, and Lehmann-Horn.[1]

Equipment/Environment

- Setup: operating room or simulation laboratory.
- Mannequin setup: Pediatric patient is intubated with a 6.0-mm cuffed endotracheal tube (ETT) under general volatile anesthesia supine with a prepared sterile field for an inguinal procedure. Patient should have a single peripheral IV access.

Props

- IV access (20 g), IV pole, 1-L normal saline bag to be used as IV fluid, noninvasive blood pressure, electrocardiogram, plethysmography, capnography, and temperature probe.
- MAC3 direct laryngoscope, 6.0-mm ID single lumen cuffed ETT, 10-cc syringe, and anesthesia machine.
- Labeled medication syringes for induction of anesthesia.
- Advanced Cardiac Life Support code cart, MH cart, and MH treatment algorithm.
- Neuromuscular stimulator and patient chart with history and physical describing no family history of MH.
- Sterile Mayo tray with basic surgical equipment.

Personnel

- Actors: nursing staff (scrub nurse/circulator; these roles may be performed by a single actor).
- Surgeon.
- Simulation learners: two individuals as anesthesia providers.
- Instructor: may be physically present to guide learners through the simulation scenario or behind the scenes, depending on the learner levels.

Prior to initiating the scenario, the multiple-choice-question pretest (Appendix D) should be administered to all learners participating. In addition to the case narrative, the patient's initial signs and symptoms of the disease process are subtle but become increasingly pronounced over the duration of the simulation to reflect MH.

After initial briefing, the simulation is activated. The patient has stable vital signs and is adequately anesthetized with isoflurane gas. The learners have a moment to get acclimated to the scenario, the anesthesia machine, and the setup. The faculty guide the learners to the vital signs and ask them to interpret them. The surgeon, who has paused during this initial introduction, then continues with the abdominal procedure. At this point, the instructor announces that 15 minutes have passed since incision.

Starting with very subtle findings, MH begins to set in on the patient. End-tidal carbon dioxide ($ETCO_2$) has gradually risen and is now at the upper limit of normal, and heart rate increases. The muscles become rigid (but this is apparent only if the patient is physically examined). The actors in the simulation act as if everything is going normally. The instructor may promote discussion but should initially give the learners time (1-2 minutes) to recognize the new findings on their own.

The vitals change again after 5 minutes, at which time the patient has a more pronounced elevation of $ETCO_2$. His temperature is at the upper limits of normal, and he has tachycardia greater than 110 bpm. Around this time, the surgeon comments that the patient's abdomen appears more rigid and asks for increased sedation. If the learners have not realized there is a suspicious process occurring, the instructor should ask them how they would respond to the surgeon's request and what medication they would administer. As the learners are talked through this process, the instructor then challenges them by pointing out various vital signs and continues to guide them as necessary, with the goal of their recognizing all the changes in vital signs and physical exam findings.

If the learners have not arrived at the appropriate diagnosis by this time (within a total of 10 minutes), the instructor explains that the patient is expressing signs and symptoms worrisome for MH, then asks the learners what the next appropriate step is. By this time, the patient's vital signs have progressively worsened. If the learners do not suggest an arterial blood gas, the instructor should encourage them to get one and to send off for some basic labs.

The instructor then guides the learners to alerting the OR staff of the high suspicion of MH. The learners should immediately inform the surgeon to discontinue the surgery as quickly as possible. At this point, the surgeon stops the procedure, and then the actor can transition to the other role of an auxiliary RN. The learners should call for help and also should call for and access the MH cart. The instructor tells one or both learners to be the leaders of the MH event. Learners should coordinate the OR staff in their respective roles and in order to complete certain tasks.

Learners should turn off all volatile anesthetics, hyperventilate the patient with high-flow 100% O_2, and instruct the OR staff to administer dantrolene as quickly as possible. The instructor provides reminders if the learners do not take appropriate steps and helps with calculations if requested by the learners. The learners should create an organized system to administer the dantrolene, delegating the tasks to mix the dantrolene and administer the mixed solution.

The simulation ends when dantrolene has been administered; supportive measures have been taken, including obtaining central venous access (or two large-bore IVs), arterial line, and cooling with cold fluids and cooling blankets; and the mannequin responds with improvement of vital signs. The learners should also direct the staff to have the patient moved to the ICU as soon as possible.

During various points in the sequence of events, critical actions must be taken in a timely manner. These include administration of a 100% FiO_2, high, fresh gas flow, hyperventilation, discontinuation of all volatile anesthetics, cooling with cold fluids and cooling blankets, requesting help and the MH cart, and organizing staff to prepare dantrolene for administration. The scenario ends when the team successfully completes the algorithm and sees an improvement in the patient's clinical condition or when time expires, whichever occurs sooner.

Debriefing

The following questions and talking points are recommended during the debriefing with the simulation learners.

MH and patient care:

- Identification of MH: The team should identify the steadily increasing $ETCO_2$, heart rate, muscle rigidity, and temperature. They should discuss the risk factors, causes, and anesthetic agents involved in MH. The team should alos discuss what questions to ask during the preoperative evaluation that may raise a red flag to indicate possible MH susceptibility.
- Management of MH: The team should alert OR staff/call for the MH cart and prepare for placement of invasive lines (large-bore IVs, arterial line, central line).
- Treatment of MH: The team should discuss the pathophysiology of MH, involving ryanodine receptors. The team should organize and set up an assembly line to reconstitute and administer dantrolene at the appropriate dose. They should discuss the mechanism of action of dantrolene and its role as treatment for MH.
- Emphasize the importance of recognizing the signs early and how intervening sooner leads to better outcomes. Talk about case reports of delayed-onset MH. In one review of published cases, patients had previous uneventful anesthesia in 20.9% of the cases, and positive family history was identified in only 24.1% of cases.[2]
- Explain that there is a designated MH expert whose information should be available on the MH cognitive aid, or as part of the MH cart, whom the learners can call at any time to help advise them in a real-life situation.

Organization and communication:

- Did the team members organize and communicate well together?
- Did they give adequate instructions and explicit tasks to individuals to perform duties?
- Did they discuss the new changes in the case with each other?

After completion of the discussions, the multiple-choice-question posttest (Appendix D) should be administered to all learners participating. Answers for each question should be discussed to ensure each learner understands the correct responses. A learner evaluation (Appendix E) of the session has also been developed and can be used by the facilitator.

Results

The simulation was completed by 24 medical students with 2 weeks of anesthesia training and daily lectures on various anesthesia topics. On average, it took 8-10 minutes before the learners began to recognize the early signs of MH. The first sign recognized was an increase in temperature, followed by the $ETCO_2$, which was generally in the high 50s before the learners recognized the possibility of MH. Most participants were hesitant to enlist the help of the OR staff in identifying the possibility of MH. No student requested the surgeon to stop the operation.

Once the signs of MH were recognized, the learners moved aggressively towards treatment options. This management by the students included the call for the MH cart and the initiation of dantrolene preparation. The last step in most of the students' management was the discontinuation of the volatile anesthetic. There were several instances where the isoflurane was maintained throughout the management of the MH. In some instances, only after prompting did the learners discover that the sustained hemodynamic collapse of the patient may have been related to the ongoing inhalation agent administration.

Most of the learners attempted to establish more secure central access or at least an additional large-bore peripheral catheter. In recognizing that the patient may have been acidotic with significant electrolyte imbalance, some of the learners also established arterial line access. In general, the students seemed to understand the need for a team-based approach to crisis management in these patients and the possibility of full circulatory collapse if MH is not promptly and adequately managed.

Verbal feedback from the learners was positive, and many students appreciated the preparation in how to prioritize the management of such a rare but life-threatening anesthesia emergency. Based on MedEdPORTAL reviewer recommendations, a learner evaluation of the session and pre- and posttest exams have been developed; however, they have not yet been used with our learners.

Discussion

The simulation curriculum was designed to educate anesthesia providers on the life-threatening disease MH. The development of the scenario we presented was created by a group of anesthesiology residents and faculty who implemented it in a simulation center at the University of Toledo Medical Center. The majority of learners were medical students rotating on the anesthesiology service over the course of several months. The results were positive, with students interacting with a simulated patient and receiving the occasional guided instruction to appropriately manage a true anesthetic emergency. Improvements to the curriculum should include properly mixing the dantrolene, which, per Harrison, Manser, Howard, and Gaba, is incorrectly mixed by up to 45% of providers in simulations.[3] Since recognition is only half the battle, treatment with a new dantrolene preparation, Ryanodex, may speed the treatment phase because of its ease of mixture. Unfortunately, not all centers have the Ryanodex formulation; therefore, it is important to learn the method of preparing the generic version for administration.

The simulation not only was received well by the students but was also crucial to understanding the benefits of simulation training in the field of anesthesiology, especially when rare diseases are difficult to encounter in real life. Future simulations will incorporate other rare but important disease processes in the simulation training environment to allow anesthesia providers to learn in a safe setting without detriment to any patient.

Johnny Quick, MD: Anesthesiology Resident, Department of Anesthesiology, University of Toledo Medical Center

Rachana Murthy: Fourth-year Medical Student, Wayne State University School of Medicine

Nitin Goyal, MD: Anesthesiology Resident, Department of Anesthesiology, University of Toledo Medical Center

Steven Margolis, MD: Anesthesiology Resident, Department of Anesthesiology, University of Toledo Medical Center

Gregory Pond, MD: Anesthesiology Resident, Department of Anesthesiology, University of Toledo Medical Center

Kimberly Jenkins, MD: Assistant Professor, Department of Anesthesiology, University of Toledo Medical Center

Disclosures
None to report.

Funding/Support
None to report.

Ethical Approval
Reported as not applicable.

References

1. Jurkat-Rott K, McCarthy T, Lehmann-Horn F. Genetics and pathogenesis of malignant hyperthermia. *Muscle Nerve*. 2000;23(1):4-17. https://doi.org/10.1002/(SICI)1097-4598(200001)23:1<4::AID-MUS3>3.0.CO;2-D
2. Strazis KP, Fox AW. Malignant hyperthermia: a review of published cases. *Anesth Analg*. 1993;77(2):297-304. https://doi.org/10.1213/00000539-199308000-00014
3. Harrison TK, Manser T, Howard SK, Gaba DM. Use of cognitive aids in a simulated anesthetic crisis. *Anesth Analg*. 2006;103(3):551-556. https://doi.org/10.1213/01.ane.0000229718.02478.c4

Received: July 14, 2016 | **Accepted:** February 16, 2017 | **Published:** March 7, 2017
