# Supplementary material for: Malignant Hyperthermia: An Anesthesiology Simulation Case for Early Anesthesia Providers
Source: MedEdPORTAL. 2017 Mar 7;13:10550. doi: 10.15766/mep_2374-8265.10550 (PMC6342051; doi:10.15766/mep_2374-8265.10550)
Supplement: Supplementary file 1 — A. Simulation Case.docx B. Critical Actions.docx C. Debriefing Materials.docx D. Pre Post Test.docx E. Simulation Course Evaluation.docx [file mep-13-10550-s001.zip › A. Simulation Case.docx]

| Appendix A: Simulation Case  SIMULATION CASE TITLE: Malignant Hyperthermia: An Anesthesiology Simulation Case for Early Healthcare Providers  AUTHORS: Johnny Quick, MD, Rachana Murthy, BS, Nitin Goyal, MD, Steve Margolis, MD, Gregory Pond, MD, Kimberly Jenkins, MD | |
| --- | --- |
| PATIENT NAME: Alvin Barnstead  PATIENT AGE: 12-year-old male  CHIEF COMPLAINT: Undergoing elective inguinal hernia repair surgery under general anesthesia | |
|  | |
| Brief narrative description of case | The patient Alvin Barnstead is a 40-kg 12-year-old Caucasian male who is undergoing an elective inguinal hernia repair. His past medical history includes asthma. His past surgical history includes circumcision at birth. He was brought to the operating room with a working 20g IV in the hand. An IV induction was performed with propofol, fentanyl, and succinylcholine. He was intubated successfully on the first attempt under direct laryngoscopy with a MAC 3 blade.  Learner goals:  Identify and treat malignant hyperthermia (MH) in the intraoperative setting. |
| Primary Learning Objectives | 1. Present a case of MH so that participants can recognize the signs and symptoms. 2. Discuss differential diagnosis of malignant hyperthermia. 3. Identify and understand the chronological order of developing MH, which includes:    1. End-tidal carbon dioxide    2. Heart rate    3. Temperature    4. Muscle rigidity 4. Identify the change in blood tests:    1. Arterial blood gas    2. Electrolyte panel    3. Renal function 5. Apply the algorithm for treatment of MH using the Malignant Hyperthermia Association of the United States (MHAUS) Emergency Therapy Protocol, including dantrolene administration, with emphasis on team work. 6. Recognize patient’s response to appropriate (and/or inappropriate) management interventions. |
| Critical Actions | 1. Alert the OR staff/Call for help 2. Call for the MH cart 3. Turn off anesthetic volatile agents and increase fresh gas flows 4. Use activated charcoal filters (Vapor-Clean™, Dynasthetics, Salt Lake City, UT) 5. Create an organized method with team members to reconstitute and administer dantrolene 6. Start 2 large bore IVs or central line, and other forms of invasive monitoring (A-line) 7. Obtain an ABG, BMP 8. Continue supportive care to keep patient hemodynamically stable and normothermic |
| Learner Preparation | Learners should have reviewed the triggering factors, signs and symptoms, and treatment protocol of malignant hyperthermia in the operating room setting. Learners will have been instructed to read the article by Jurkat-Rott, McCarthy and Lehmann-Horn. |

| Initial Presentation | | | |
| --- | --- | --- | --- |
| Initial vital signs | HR 80, BP 115/75, Temp(C) 37, etCO2 38, SaO2 99%, RR 11, WT 40-kg | | |
| Overall Appearance | The patient is intubated with a 6.0 mm ID cuffed endotracheal tube, with a peripheral 20g IV with normal saline. The surgical drape is attached to two IV poles, and the surgeon is operating in the inguinal region on the patient. The anesthesia machine and cart are at the head of the bed and a vital sign monitor is displaying the patient’s heart rate, blood pressure and end-tidal CO2. | | |
| Actors and roles in the room at case start | Simulation Learners (at least 2 individuals in an anesthesia provider role)  Nursing Staff (Scrub Nurse/Circulator; these roles may be performed by a single actor)  Surgeon | | |
| HPI | The patient Alvin Barnstead is a 40-kg 12-year-old Caucasian male who is undergoing an elective inguinal hernia repair. His past medical history includes asthma, with rare attacks occurring during winter months. He had been circumcised at birth, and has no family history of anesthesia related complications. He is an active child, and eats a healthy, well balanced diet. In the preoperative phase, he was given oral midazolam and a 20g IV in the right hand was placed without difficulty. He was then brought to the operating room and an IV induction was performed with propofol, fentanyl, and succinylcholine. He was intubated successfully on the first attempt under direct laryngoscopy with a MAC 3 blade. The scrub tech has just finished setting up the sterile field, and the procedure is about to begin.  The role of the learner is to manage the anesthetic requirements and monitor the vital signs during the operation. The learner should identify any abnormalities consistent with malignant hyperthermia and treat them accordingly during this time. | | |
| Past Medical/Surgical History | Medications | Allergies | Family History |
| Asthma, controlled with less than 2 attacks/month, triggered by cold weather  Surgical history: circumcision | Albuterol inhaler for wheezing/bronchospasm | none | parents deny any history of adverse events from anesthesia |
| Physical Examination | | | |
| General | Sedated, male child | | |
| HEENT | WNL with a 6.0 mm ID cuffed endotracheal tube orally placed and secured with tape | | |
| Neck | WNL | | |
| Lungs | Clear and equal breath sounds bilaterally, no wheezing | | |
| Cardiovascular | Regular rate and rhythm, no murmurs | | |
| Abdomen | Covered with sterile drapes, no identifiable abnormalities | | |
| Neurological | Patient is anesthetized, unable to obtain further information | | |
| Skin | WNL | | |

| Instructor Notes - Changes and CASE Branch Points | | |
| --- | --- | --- |
| Intervention / Time point | Change in Case | Additional Information |
| 3 minutes into the case | Instructor will inform the learner 15 minutes have passed since incision. Vital signs now show HR 105, BP 132/90, Temp 37.7 C, ETCO2 45, SaO2 98%, and RR 16, with muscle rigidity on physical exam |  |
| 1-2 minutes after first vital signs changes | Vital signs gradually increase to HR 120, BP 149/101, Temp 38.4 C, ETCO 54, SaO2 95%, RR 25, and surgeon mentions muscle rigidity in the surgical field. | Instructor will point out vital sign changes if not apparent to learners and ask how the learner plans to respond to the new changes. |
| 5-8 minutes after initial change in vital signs |  | Learner should already suspect MH. If learners have not reached this possibility, instructor inform them of the likely diagnosis of malignant hyperthermia |
| Learner requests an ABG and other labs | ABG shows pH 7.25, pCO2 55, pO2 95, HCO3 16. |  |
| Learner alerts OR staff to high suspicion of MH and calls for help and for the MH cart to be brought into room | MH cart is brought into room, and additional staff arrives at room. | Instructor has one learner become leader for organizing roles in MH protocol and preparation of dantrolene. |
| Learner should turn off all volatile agents and administer 100% FiO2 |  | Instructor should remind learners if not already performed. |
| Dantrolene is administered to patient at 2.5mg/kg | Vital signs should start to stabilize and return to normal values. |  |
| Additional IV access should be attained or attempted by learners |  |  |

Ideal Scenario Flow

The learners enter the room to an anesthetized patient with vital signs displayed on a monitor, including heart rate, blood pressure, and end-tidal CO2. A surgeon is operating on the inguinal area. Initial vital signs are normal and stable. The surgeon proceeds with the surgery and after the instructor announces 15 minutes have passed since incision, the learners will notice the ETCO2 gradually rising and heart rate increasing. They immediately recognize malignant hyperthermia a likely diagnosis and order an ABG, alert the OR staff, and request the surgeon to stop the surgery at a non-critical point. The learners will turn the volatile anesthetic off, start delivery 100% FiO2, request the MH cart be brought to the room and have the OR staff start preparing dantrolene. They will administer the dantrolene at 2.5mg/kg and continue monitoring the patient for signs of improvement. Once stable, the patient is sent to the ICU for further supportive care with serial blood gases and urine measurements to be monitored for resolution of malignant hyperthermia.

Anticipated Management Mistakes

1. Failure to recognize the diagnosis of MH: We realized that many learners did not immediately arrive at the possible diagnosis of malignant hyperthermia. If this happened, the instructor would have the vital signs changed to a worsening stage of MH with temperature rising with tachycardia and hypercarbia and the surgeon would note muscle rigidity in the surgical field. If the learners still do not realize the diagnosis, the instructor will explain the patient is expressing signs and symptoms consistent with MH and ask the learner for the next step in management.
2. Failure to recognize the need for the MH cart and dantrolene: Some of learners were unaware of the need to treat MH with dantrolene and that the MH cart would be necessary to prepare and administer the treatment. If this occurred, the instructor would help guide the learners to organize the OR staff and call for the cart to prepare the treatment.
3. Failure to recognize the need to turn off volatile anesthetics: Some learners did not recognize the need to turn the offending agent, the volatile anesthetic, off and administer 100% FiO2. If this occurred, the patient would continue to worsen until the learner recognized this step or the instructor intervened and informed the learner to turn the agent off.
