# Supplementary material for: Malignant Hyperthermia: An Anesthesiology Simulation Case for Early Anesthesia Providers
Source: MedEdPORTAL. 2017 Mar 7;13:10550. doi: 10.15766/mep_2374-8265.10550 (PMC6342051; doi:10.15766/mep_2374-8265.10550)
Supplement: Supplementary file 1 — A. Simulation Case.docx B. Critical Actions.docx C. Debriefing Materials.docx D. Pre Post Test.docx E. Simulation Course Evaluation.docx [file mep-13-10550-s001.zip › B. Critical Actions.docx]

Appendix B:

Critical Actions List

A. The following actions should be performed in a timely manner by the learners:

1. Recognize signs and symptoms that something is wrong with the patient (i.e., aberrant vital signs, muscle rigidity) and alert OR team that MH is suspected.
2. Discontinue offending agent, hyperventilate with high flow and 100% FiO2.
3. Identify critical changes and implement treatment accordingly.

B. Each of the following actions are required in management of the patient:

1. Discontinue all volatile anesthetics.

2. Administer 100% FIO2 at high flows.

3. Hyperventilate the patient.

4. Call for help and for the MH cart.

5. Order basic labs and ABG.

6. Initiate various forms of cooling (chilled saline; gastric lavage, discontinuation of warming devices).

7. Prepare and administer dantrolene.
