# Supplementary material for: Malignant Hyperthermia: An Anesthesiology Simulation Case for Early Anesthesia Providers
Source: MedEdPORTAL. 2017 Mar 7;13:10550. doi: 10.15766/mep_2374-8265.10550 (PMC6342051; doi:10.15766/mep_2374-8265.10550)
Supplement: Supplementary file 1 — A. Simulation Case.docx B. Critical Actions.docx C. Debriefing Materials.docx D. Pre Post Test.docx E. Simulation Course Evaluation.docx [file mep-13-10550-s001.zip › C. Debriefing Materials.docx]

Debriefing:

The following questions and talking points are recommended during the debriefing with the simulation learners.

- 1. Malignant Hyperthermia and Patient Care:
     1. Identification of malignant hyperthermia. Team should identify the steadily increasing end tidal CO2, heart rate, muscle rigidity, and temperature. Team should discuss the risk factors, causes, and anesthetic agents involved in MH. The team should discuss what questions to ask during the preoperative evaluation that may raise a red flag to indicate possible MH susceptibility.
     2. Management of malignant hyperthermia. Team should alert OR staff/call for help and for the MH cart, and prepare for placement of invasive lines (large bore IVs, arterial line, central line).
     3. Treatment of malignant hyperthermia. Team should discuss the pathophysiology of MH, involving ryanodine receptors. Team should organize and set up an assembly line to reconstitute and administer dantrolene at the appropriate dose. Team should discuss the mechanism of action of dantrolene and its role as treatment for MH.
     4. Emphasize the importance of recognizing the signs early, and intervening early leads to better outcomes. Talk about case reports of delayed onset MH. In one review of published cases, patients had previous uneventful anesthesia in 20.9% of the cases and positive family history was only identified in 24.1% of cases (Anesth Analg 1993;77:297-304).
     5. Explain that there is a designated MH expert whose information should be available on the MH cognitive aid, or as part of the MH cart, whom the learners can call at any time to help advise them. Malignant Hyperthermia Hotline: 1-800-644-9737
  2. Organization and Communication:
     1. Did the team organize and communicate well together?
     2. Did they give adequate instructions and explicit tasks to individuals to perform duties?
     3. Did they discuss the new changes in the case with each other?
