# Supplementary material for: Malignant Hyperthermia: An Anesthesiology Simulation Case for Early Anesthesia Providers
Source: MedEdPORTAL. 2017 Mar 7;13:10550. doi: 10.15766/mep_2374-8265.10550 (PMC6342051; doi:10.15766/mep_2374-8265.10550)
Supplement: Supplementary file 1 — A. Simulation Case.docx B. Critical Actions.docx C. Debriefing Materials.docx D. Pre Post Test.docx E. Simulation Course Evaluation.docx [file mep-13-10550-s001.zip › D. Pre Post Test.docx]

**Appendix D: MCQ Pretest/Posttest**

1. Malignant hyperthermia is triggered by:
   1. Desflurane
   2. Isoflurane
   3. Halothane
   4. Sevoflurane
   5. All of the above
2. The earliest sign(s) of malignant hyperthermia is(are):
   1. Tachycardia
   2. Rising end-tidal CO2 (EtCO2)
   3. Elevated Temperature
   4. Rhabdomyolysis
   5. Both A & B
3. The treatment of choice for MH is:
   1. Epinephrine
   2. Dantrolene
   3. Vasopressin
   4. Nitroglycerin
   5. None of the above
4. The most common protein mutation in cells that results in MH is in the:
   1. Sodium-potassium ATPase
   2. Sodium-Glucose transporter
   3. Acetylcholine receptor
   4. Ryanodine receptor
5. Of the following, which inhalational agent is considered safe to use in an MH-susceptible patient?
   1. Desflurane
   2. Ether
   3. Halothane
   4. Nitrous Oxide
   5. None of the above are safe for use
