# Supplementary material for: Malignant Hyperthermia: An Anesthesiology Simulation Case for Early Anesthesia Providers
Source: MedEdPORTAL. 2017 Mar 7;13:10550. doi: 10.15766/mep_2374-8265.10550 (PMC6342051; doi:10.15766/mep_2374-8265.10550)
Supplement: Supplementary file 1 — A. Simulation Case.docx B. Critical Actions.docx C. Debriefing Materials.docx D. Pre Post Test.docx E. Simulation Course Evaluation.docx [file mep-13-10550-s001.zip › E. Simulation Course Evaluation.docx]

**Appendix E: Simulation Course Evaluation**

Please evaluate this session by indicating your agreement with the following statements [5 = Strongly agree, 4 = Agree, 3 = Neither agree nor disagree, 2 = Disagree, 1 = Strongly Disagree]:

| This experience will improve my performance in actual clinical settings. |  |
| --- | --- |
| This simulation was a valuable learning experience. |  |
| This debriefing was a valuable learning experience. |  |
| The objectives of this simulation were met. |  |
| I would recommend this simulation to others |  |
| The length of time for the simulation was appropriate |  |
| The length of time for debriefing was appropriate |  |

Two things I enjoyed/learned from the simulation:

Two things that could have been improved or more focused upon:
